# Guillain-Barré Syndrome After a SARS-CoV-2 Vaccine

**DOI:** 10.7759/cureus.57705

**Published:** 2024-04-06

**Authors:** Ana M Oliveira, Patrícia Varela Ramos, Gonçalo Durão-Carvalho, Vânia Almeida, João Gonçalves Pereira

**Affiliations:** 1 Intensive Care Unit, Hospital de Vila Franca de Xira, Vila Franca de Xira, PRT; 2 Internal Medicine, Centro Hospitalar do Oeste, Unidade de Caldas da Rainha, Caldas da Rainha, PRT; 3 Neurology, Hospital de Vila Franca de Xira, Vila Franca de Xira, PRT

**Keywords:** adverse reactions, neuroinflammatory severe complications, surveillance, sars-cov-2 vaccine, guillain-barré syndrome (gbs)

## Abstract

The worldwide mass vaccination campaign against COVID-19 has been the largest one ever undertaken. Although the short-term safety profile of the different vaccines has been well established, neuroinflammatory complications have been described, including rare cases of acute demyelinating inflammatory polyneuropathy.

We report a 63-year-old man who presented to the emergency department with proximal muscle weakness and paresthesia. He had received the first dose of the AZD1222 SARS-CoV-2 vaccine (Oxford, AstraZeneca) two weeks before presentation. The diagnosis of Guillain-Barré syndrome (GBS) was confirmed by clinical features, cerebrospinal fluid analysis, and electromyography. On the second hospital day, progression to flaccid tetraplegia, cranial nerve involvement, and respiratory failure, requiring invasive mechanical ventilation, were noted, and he was admitted to the intensive care unit. Despite the prompt diagnosis and immunosuppressive treatment initiation, the patient was left with severe disability.

Although the COVID-19 vaccination was generally safe and socially beneficial, individual adverse reactions, including neuroinflammatory severe complications, may occur.

## Introduction

The first case of severe acute respiratory syndrome caused by coronavirus 2 (SARS-CoV-2) was reported in Wuhan, China, in December 2019. Two months later, the coronavirus disease 2019 (COVID-19) worldwide pandemic was declared, and vaccination was established as a social priority. The COVID-19 pandemic drastically affected human life, with a mortality rate of 3.4% at the early stages [1]. Although COVID-19 disease predominantly causes a respiratory illness [2], systemic complications may occur, including central and peripheral nervous system involvement [3,4], such as the Guillain-Barré syndrome (GBS) [5].

After the approval of the first COVID-19 vaccine in December 2020, one of the largest worldwide mass vaccination campaigns was undertaken [6]. In clinical trials, several vaccine side effects have been reported, ranging from mild symptoms (mostly myalgia, fatigue, and fever) to more severe ones, like anaphylactic shock [6]. Acute neuroinflammatory complications have also been described, including rare cases of GBS [6,7].

GBS is an immune-mediated polyradiculoneuropathy presenting with a variable combination of rapid progressive weakness, sensory disturbances, cranial nerve involvement, and autonomic instability. A careful history usually reveals an antecedent infection, mainly upper respiratory or gastrointestinal, or other antecedent events like vaccination, surgery, or drugs. The diagnosis is based on clinical evaluation, electromyography, and cerebrospinal fluid analysis. Most cases are self-resolving, but some patients experience life-threatening respiratory muscle paralysis requiring mechanical ventilation.

We present a case report of GBS temporally associated with the first dose of the AZD1222 SARS-CoV-2 vaccine to increase awareness of acute inflammatory demyelinating polyradiculoneuropathy (AIDP) as a possible side effect of the COVID-19 vaccine.

## Case presentation

A 63-year-old man presented to the emergency department in July of 2021 with acute proximal tetraparesis, more severe in the lower limbs, intense calf myalgia, diffuse paresthesia, and hypophonia that progressed in less than 24 hours. His past medical history was remarkable for secondary progressive multiple sclerosis (MS), with long-term stable proximal paraparesis and dysarthria (Expanded Disability Status Scale of 6.5). Disease-modifying therapy had been discontinued for months due to a lack of efficacy. Arterial hypertension and a myocardial infarction in the past were also reported. He had received his first dose of the AZD1222 SARS-CoV-2 vaccine (Oxford, AstraZeneca) two weeks before presentation. He denied any history of recent infections or other medical procedures. He also denied a past COVID-19 infection.

Physical examination revealed a pronounced hypophony, with imperceptible speech, hypomotility of the palate veil, shortness of breath with desaturation, hypotonic tetraparesis, with a predominance of proximal weakness on both upper and lower limbs, and bilateral weak osteotendinous reflexes. No other changes were appreciated. 

**Figure 1:**
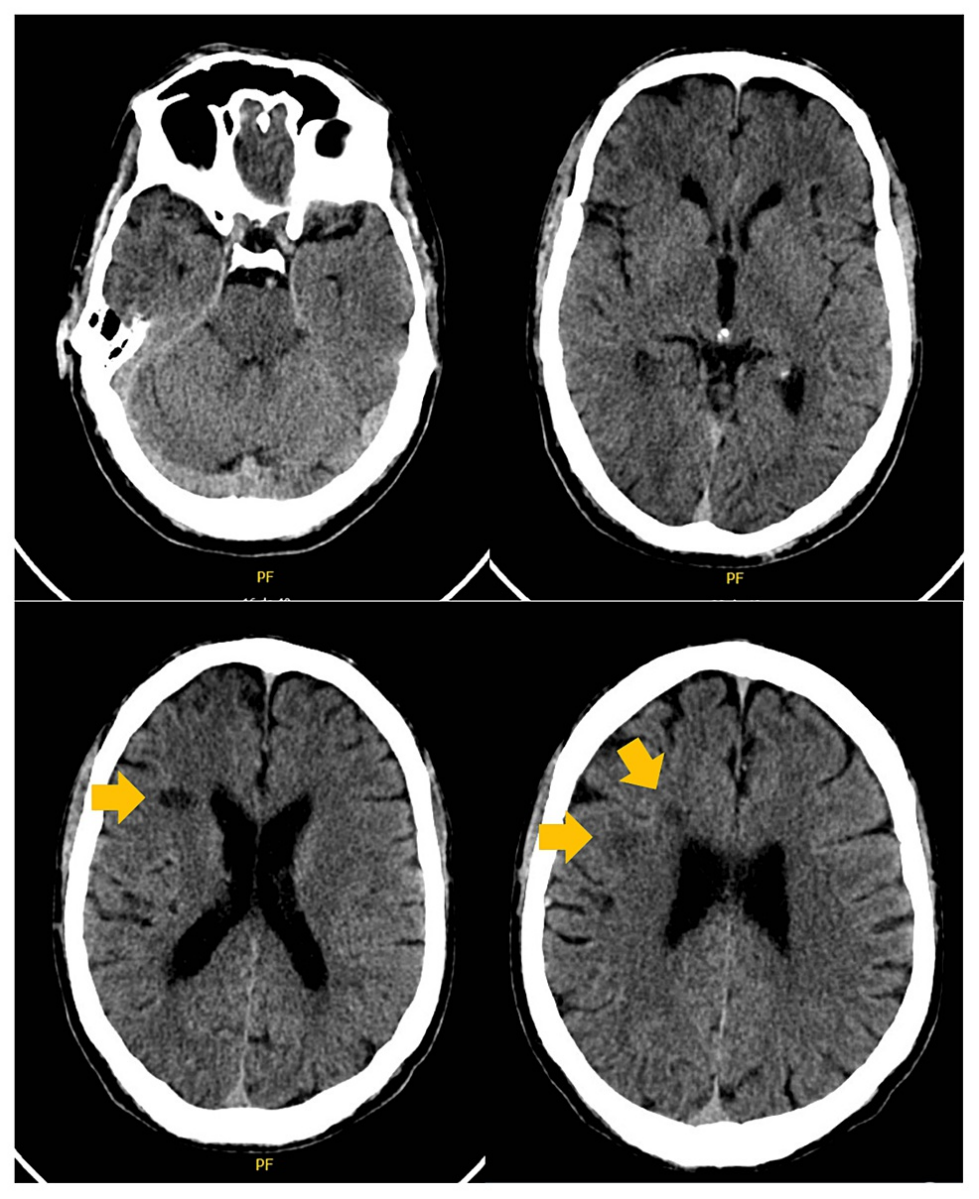
A brain CT scan without contrast showed multiple subcortical hypodense lesions without mass effect, compatible with the past medical history of multiple sclerosis. No mass lesions, hemorrhage, hydrocephalus, or extra-axial collections were found.

A brain CT scan (Figure 1), performed at admission, disclosed multiple diffuse white matter hypoattenuating lesions compatible with plaques from MS. Blood tests were unremarkable, including viral serological tests (Table 1). A SARS-Cov-2 polymerase chain reaction (PCR) test was negative. Initially, an MS relapse was considered, and the patient was given methylprednisolone 1 g/day.

**Table 1:** Complementary diagnostic tests performed in the emergency department. MCV: mean corpuscular volume; MCH: mean corpuscular hemoglobin; MCHC: mean corpuscular hemoglobin concentration; PT: prothrombin time; INR: international normalized ratio; APTT: activated partial thromboplastin time; AST: aspartate aminotransferase; ALT: alanine aminotransferase; GGT: gamma-glutamyl-transpeptidase; ALP: alkaline phosphatase; LDH: lactate dehydrogenase; CRP: C-reactive protein; hs-cTn: high-sensitivity troponin I; Ab: antibodies; CMV: cytomegalovirus; Ig: immunoglobulin; EBV: Epstein-Barr virus; PCR: polymerase chain reaction; RDW-CV: red cell distribution width-coefficient of variation

Blood count		
	Results	Normal range
Hemoglobin	15.8 g/dL	13.0-17.0
Hematocrit	44.9 %	40.0-50.0
MCV	85.5 fL	80.0-97.0
MCH	30.1 pg	27.0-32.0
MCHC	35.2 g/dL	32.0-36.0
RDW-CV	12.8 %	11.6-14.0
Leukocytes	12.6x10^3 ^/μL	4.0-10.0
Neutrophils	11.52x10^3 ^/μL	1.5-8.0
Eosinophils	0.00x10^3 ^/μL	0.0-0.3
Basophils	0.01x10^3 ^/μL	0.0-0.3
Lymphocytes	0.74x10^3 ^/μL	0.8-4.0
Monocytes	0.33x10^3 ^/μL	0.0-1.2
Platelets	189x10^3 ^/μL	150-400
Coagulation tests		
PT	12.0 seg.	11-13
INR	1.00	-
APTT	25 seg.	22.1-28.1
D-Dimers	1622.00 ng/mL	Cut-off: 500 ng/mL
Biochemistry		
Serum creatinine	0.63 mg/dL	0.70-1.30
Serum urea	43 mg/dL	<50
Sodium	133 mmol/L	136-145
Potassium	4.31 mmol/L	3.5-5.1
Chlorine	97 mmol/L	98-107
Phosphate	2.8 mg/dL	2.5-4.9
Calcium	9.2 mg/dL	8.5-10.1
Magnesium	1.9 mg/dL	1.8-2.4
AST	16 UI/L	15-37
ALT	32 UI/L	16-63
GGT	24 UI/L	15-85
ALP	80 UI/L	50-136
Total bilirubinemia	0.96 mg/dL	<1
Total creatine kinase	87 UI/L	39-308
LDH	186 UI/L	85-227
Albumin	3.75 g/dL	3.4-5
CRP	4,46 mg/dL	0.06-1.00
hs-cTn I	52.9 pg/mL	<72.0
Folic acid	4.6 ng/mL	>5.38
B12 vitamin	423.0 pg/mL	211-911
Serological tests		
Ab. Anti-CMV	IgG positive; IgM negative	
Ab. Anti-EBV	IgG positive; IgM negative	
Herpes simplex (1 and 2)	IgG positive; IgM negative	
Mycoplasma pneumonia	IgG positive; IgM negative	
Other tests		
PCR SARS-CoV-2	Negative	

**Figure 2:**
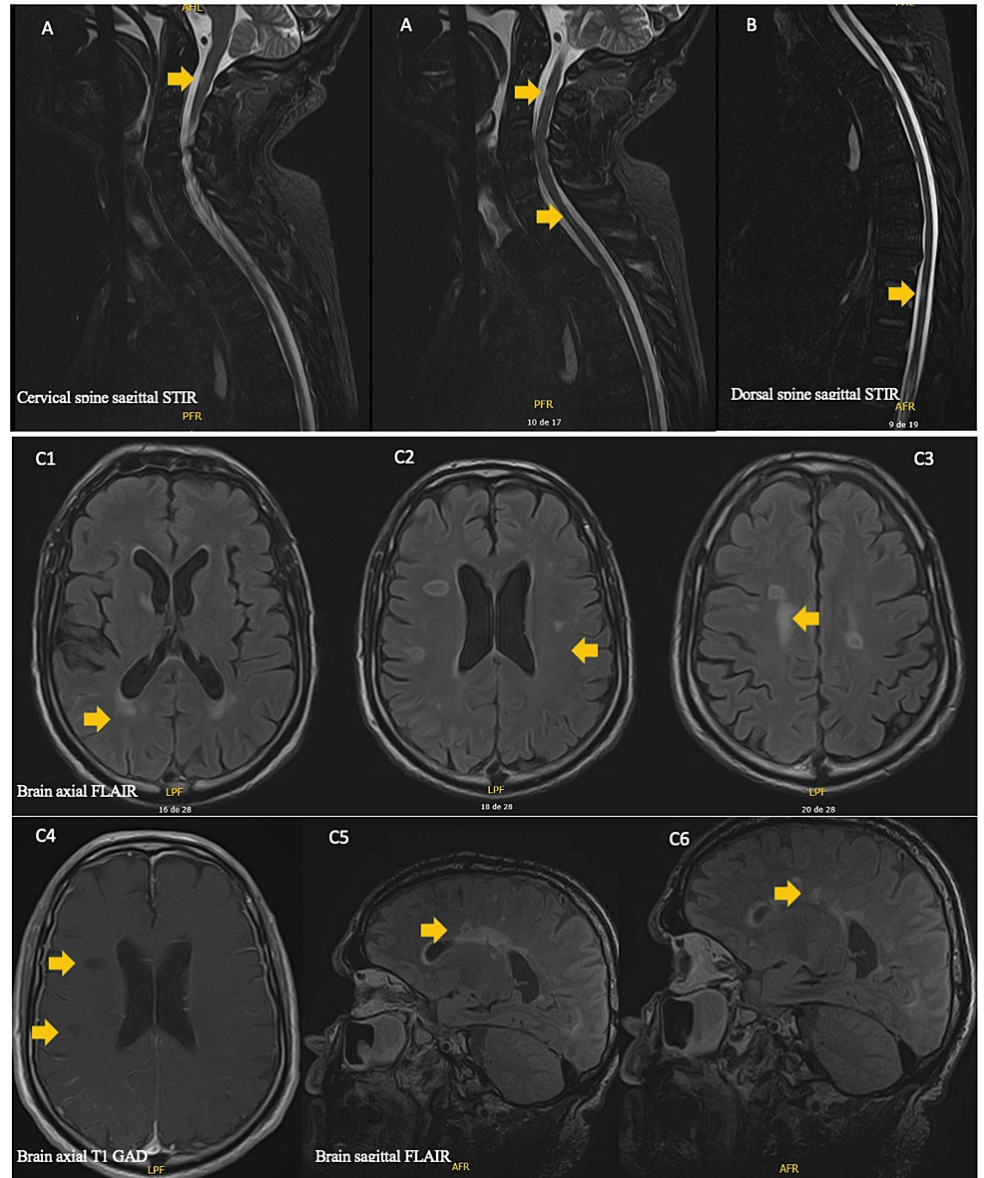
MRI showing multiple cervical (A), dorsal (B), and brain periventricular (C1), deep (C2), and juxtacortical (C3) white matter lesions without contrast-enhancing (C4). T1 black holes are indicative of a chronic stage with white matter destruction, axonal loss, and an irreversible clinical outcome (C4). Periventricular demyelinating plaques extending radially away from the body of the lateral ventricle are characteristic of multiple sclerosis (C5-6).

In the first hours after admission, the progression of neurologic deficits to severe areflexic flaccid tetraparesis, anarthria, dysphagia, bilateral facial palsy, and respiratory failure were noted. A GBS was considered, and he was admitted to the intensive care unit (ICU). Cerebrospinal fluid examination revealed albuminocytologic dissociation (total protein 122.70 mg/dL and white blood cells 40/uL, Table 2). Methylprednisolone was discontinued, and intravenous immunoglobulin was started (0.4 g/kg daily for five days). Despite the clinical efforts, global respiratory failure, and the need for invasive mechanical ventilation (IMV) supervene. Autonomic dysfunction was also observed. An electromyographic examination performed six days after clinical presentation confirmed a severe acquired acute demyelinating polyneuropathy (Table 3). Magnetic resonance imaging of the brain and the spine showed the already-known white matter plaques, including in the prefrontal, periventricular, deep white matter, juxtacortical, brainstem, and high cervical areas, without contrast enhancement (Figure 2). In the next few days, no clinical improvement was noted, despite intravenous immunoglobulin. Plasmapheresis (plasma exchange 200-250 ml/kg) was performed for five consecutive days.

**Table 2:** Cerebrospinal fluid analysis. Ig: immunoglobulin; CSF: cerebrospinal fluid

Cerebrospinal fluid		
	Results	Normal range
Aspect	Clear	-
Nº Nucleated cells	40 Cel/μL	-
Erythrocytes	0.00x10^6^ /μL	-
Leukocytes	40 Cel/μL	<5
Polymorphonuclear	27 Cel/μL (68%)	-
Monomorphonuclear	13 Cel/μL	-
Glucose	95 mg/dL	>40
Proteins	122.70 mg/dL	15.0-45.0
Albumin	80.0 mg/dL	8.0-300.0
IgG	10.6 mg/dL	<3.4
Oligoclonal bands	Negative	
Blood		
IgG	896.9 mg/dL	700.0-1600.0
Albumin	4.1 g/dL	3.4-5.0
CSF immunoglobulin G (IgG) index	0.6	<0.7

**Table 3:** Summary of electromyography and nerve conduction data. ABP: abductor pollicis brevis; ADM: abductor digiti minimi; AH: abductor hallucis; EDB: extensor digitorum brevis; MUP: motor unit potencial; R: right

Motor nerve conduction studies						
Nerve	Stimulation site	Recording site	Distal motor latency (ms)	Amplitude (mV)	Conduction velocity (m/s)	H reflex (ms)
R Median	Wrist	APB	10.08 (n < 4.0)	0.04 (n > 4)	-	-
	Antecubital fossa	APB	-	0 (n > 4)	0 (n > 49)	-
R Ulnar	Wrist	ADM	6.27 (n < 3.6)	0.12 (n > 5)	-	-
	Below elbow	ADM	-	0 (n > 5)	0 (n > 50)	-
R Peroneal	Ankle	EDB	0 (n < 6.2)	0 (n > 2)	-	-
	Below knee	TA	7.96 (n ≤ 6.7)	0.05 (n > 4)	-	-
	Above knee	TA	-	0.02 (n > 4)	27.5 (n > 44)	-
R Tibial	Ankle	AH	8.14 (n < 6)	0.66 (n > 3)	-	-
	Knee	AH	-	0.07 (n > 3)	29.8 (n > 39)	Absent (n < 35)
Sensory nerve conduction studies						
Nerve	Stimulation site	Recording site	Latency (ms)	Amplitude (uV)	Conduction velocity (ms)	-
R Median	Wrist	Digit 3	3.7 (n < 3.5)	0.73 (n > 15)	44.6 (n > 50)	-
R Radial	Forearm	Snuff box	2.47 (n > 2.2)	3.1 (n > 15)	57.5 (n > 50)	-
R Superfi cial peroneal	Above lateral malleolus	Dorsum of foot	0 (n > 4)	0 (n > 4)	0 (n > 40)	-
R Sural	Calf	Lateral malleolus	2.94 (n > 3.1)	5.5 (n > 5)	49.3 (n > 40)	-
Needle electromyography						
Muscle	Insertional activity	Spontaneous activity	Activation	Recruitment	MUP morphology	Interferential pattern
R deltoid	Normal	0	None	-	-	0 activity
R biceps brachii	Normal	0	None	-	-	0 activity
R tibialis anterior	Normal	0	None	-	-	0 activity

His ICU stay was also complicated by ventilator-associated pneumonia, acute bleeding diatheses, and inappropriate antidiuretic hormone secretion syndrome. Both a tracheostomy and a percutaneous gastrostomy were placed. After five months, he was discharged to an acute rehabilitation facility with a slight improvement in proximal upper limb strength, still under ventilatory support, and a percutaneous gastrostomy tube.

## Discussion

We present a case of severe GBS that developed after 15 days of the first dose of the SARS-CoV2 vaccination. Rapid deterioration was noted, with the need for invasive ventilatory and vasopressor support. Despite early immunosuppressive treatment, the recovery was very slow and incomplete. The presence of a previous neurologic motor disease may have put this patient at special risk for a more severe form of vaccine-associated GBS.

GBS is an acute immune-mediated generalized inflammatory polyradiculoneuropathy with an annual global incidence of approximately one to two per 100,000 person-years [1], more frequent in males and older patients [1,8]. GBS typically presents with rapidly progressive, frequently ascending, symmetrical motor weakness and paresthesia, with hyporeflexia or areflexia, resulting in severe and sometimes lasting paralysis [9,10]. Cranial nerve deficits, respiratory failure, and autonomic dysfunction can also be present [11]. Even though the exact pathophysiology is unknown, GBS is believed to be caused by an aberrant immune-mediated response resulting from the generation of autoimmune antibodies that cross-react with epitopes on peripheral nerves, leading to nerve damage [8,10]. Most patients report a precipitant event up to four weeks before weakness develops, mainly Campylobacter jejunii infections [8,12]. Moreover, it has also been linked with previous vaccination, surgery, and immune checkpoint inhibitor therapy [8].

Although the timeline in our patient strongly suggests a link between COVID-19 vaccination and GBS, so far, the influenza vaccine was the only one with an established causal association with GBS, namely the 1976 A/New Jersey influenza (‘swine flu’) vaccination (with an incidence of approximately one in 100,000 vaccinations) [8]. Subsequent studies failed to show conclusive evidence of a causal association between GBS and most vaccines. Currently, the risk of developing GBS with other influenza vaccines is estimated to be inferior to one per million vaccinations [8,10]. The COVID-19 pandemic outbreak fostered worldwide mass vaccination campaigns [5,9]. As of late November 2020, four vaccines have been approved [1,9]. Many side effects were reported, ranging from fatigue, fever, and myalgia to more serious complications. An increased risk of GBS after vaccination with adenovirus-vectored COVID-19 vaccines (ChAdOx1 nCoV‐19 (Oxford‐AstraZeneca)) and Ad.26.COV2.S (Janssen) has been reported [13], although the same has been disputed [1,14]. The prevalence of GBS after vaccination against COVID-19 has been established at 8.1 per 1,000,000 vaccinations, significantly higher than GBS in the general population [11]. Almost all case reports of GBS after COVID-19 vaccination occurred after the first dose, like in our patient [14,15].

The primary pathogenic mechanism for vaccine-associated GBS is not completely understood. It has been proposed that the acute immune response induced by the vaccine can also trigger an autoimmune process with antibodies that may cross-react, through molecular mimicry, with glycoproteins on the myelin sheath of the axons of peripheral nerves and lead to GBS [9,10,11]. Adenovirus-vectored COVID-19 vaccines are recombinant vaccines that use a nonreplicating adenovirus vector encoding the SARS-CoV-2 spike protein to trigger an immunologic antibody response [13,16]. Specifically, immune cross-reactivity, triggered by the similarity between vaccine components and peripheral nerve components (gangliosides), could cause the immune system to attack similar proteins. Accordingly, vaccinated people could produce anti-ganglioside autoantibodies that attack neural antigens, causing neurological damage [13,16,17]. Other phenomena, namely the production of specific autoantibodies and the role of certain vaccine adjuvants, seem to be substantial contributors to this autoimmune phenomenon [16]. An association with facial (Bell’s) palsy was also reported among neurological complications after COVID-19 vaccination, particularly when adenovirus-vectored COVID-19 vaccines were used [1,13,16]. Nevertheless, these processes have not been described for mRNA COVID-19 vaccines, either BNT162b2 (Pfizer-BioNTech) or mRNA-1273 (Moderna) [11,17].

Of all criteria for assessing the causal relationship between clinical outcome and a possible adverse event of COVID-19 vaccines, only temporality was found to be relevant [13]. Expert consensus, largely derived from vaccination surveillance, considers a six-week period for a vaccine-attributable risk of GBS [14]. A peak between 11 and 22 days after vaccination is reported, which is the time point when the vaccine promotes its maximum immune response [13,16]. Our patient had a clear temporal relationship between receiving the vaccine and developing muscle weakness. Nevertheless, there are no specific biological markers that can prove this causality [10].

Conversely, GBS has also been associated with SARS-CoV-2 infection, even with a higher reported incidence than post-vaccination GBS [18], which reinforces the advantage of the vaccination campaign.

Overall, GBS is usually associated with a favorable outcome, with most patients experiencing stabilization until four weeks and significant improvement afterward [10], and this has also been reported in COVID-19-associated GBS [19]. No specific better or worse prognosis has been associated with vaccine-induced GBS. The early need for IMV, severe weakness at nadir, and rapid onset of weakness, as noted in our patient, have been identified as adverse prognostic factors. Cardiac arrhythmias and blood pressure instability can occur in 3-10% of patients with GBS, even after receiving plasmapheresis or intravenous immunoglobulin (IVIG) therapy [9,10], owing to the involvement of the autonomic nervous system [10]. Of note, about 40% of patients treated with IVIG or plasmapheresis do not improve within the first four weeks after treatment, and severe motor impairments persist in more than 10% of cases, with a prolonged course of ventilator dependency and a very delayed and incomplete recovery [1], as noted in our patient. GBS presents a mortality rate of 3-7% despite intensive care, and among patients who become ventilator-dependent, the mortality rate can increase up to 20% [20].

Despite these adverse events, dramatic reductions in severe illness, hospitalizations, and death have been achieved with mass vaccination [9,12]. Unveiling the epidemiology of rare vaccine complications is critical to preventing unnecessary morbidity and mortality [9]. Rigorous surveillance and notification of all potential adverse effects of vaccines must be carried out for a more rigorous knowledge of their safety profile.

## Conclusions

We report a severe case of GBS after the AZD1222 SARS-CoV-2 vaccine. The patient had a previous significant motor neurological disease that may have facilitated the cross-reaction of antibodies against COVID-19 with myelin sheath glycoproteins and led to a more severe GBS and persistent disease.

Although vaccination is associated with significant social benefits that largely outweigh its risks, serious adverse reactions can occur. Early diagnosis and treatment of neurological complications, especially in at-risk patients, are critical. Surveillance and notification are of utmost importance to increase knowledge of its safety profile, unveil risk factors for complications, and help prevent individual morbidity and mortality.
